# Longer Telomeres Are Associated with Cancer Risk in MMR-Proficient Hereditary Non-Polyposis Colorectal Cancer

**DOI:** 10.1371/journal.pone.0086063

**Published:** 2014-02-03

**Authors:** Nuria Seguí, Elisabet Guinó, Marta Pineda, Matilde Navarro, Fernando Bellido, Conxi Lázaro, Ignacio Blanco, Victor Moreno, Gabriel Capellá, Laura Valle

**Affiliations:** 1 Hereditary Cancer Program, Catalan Institute of Oncology, IDIBELL, Hospitalet de Llobregat, Barcelona, Spain; 2 Unit of Biomarkers and Susceptibility, Catalan Institute of Oncology, IDIBELL and CIBERESP, Hospitalet de Llobregat, Barcelona, Spain; 3 Department of Clinical Sciences, Faculty of Medicine, University of Barcelona, Hospitalet de Llobregat, Barcelona, Spain; Ohio State University Medical Center, United States of America

## Abstract

Aberrant telomere length measured in blood has been associated with increased risk of several cancer types. In the field of hereditary non-polyposis colorectal cancer (CRC), and more particularly in Lynch syndrome, caused by germline mutations in the mismatch repair (*MMR*) genes, we recently found that cancer-affected *MMR* gene mutation carriers had shorter telomeres and more pronounced shortening of telomere length with age than controls and unaffected *MMR* gene mutation carriers. Here we evaluate blood telomere length in MMR-proficient hereditary non-polyposis CRC, i.e. familial CRC type X (fCRC-X). A total of 57 cancer-affected and 57 cancer-free individuals from 34 Amsterdam-positive fCRC-X families were analyzed and compared to the data previously published on 144 cancer-affected and 100 cancer-free *MMR* gene mutation carriers, and 234 controls. Relative telomere length was measured using a monochrome multiplex quantitative PCR method, following strict measures to avoid sources of bias and adjusting by age. Despite the retrospective nature of our study, the results show that longer telomeres associate with cancer risk in fCRC-X, thus identifying different patterns of telomere length according to the status of the MMR system.

## Introduction

Family history is one of the strongest risk factors for the development of colorectal cancer (CRC) and is involved in approximately 20% of all CRC cases. However, only 2–6% of all CRCs are explained by germline mutations in known high-penetrance CRC genes. The Amsterdam criteria were defined to identify hereditary non-polyposis CRC cases, considering young age (<50 years) at cancer diagnosis and high familial aggregation of CRC (Amsterdam I) or other related tumors (Amsterdam II). Approximately 60% of the families that fulfill the Amsterdam criteria show DNA mismatch repair (MMR) deficiency as a consequence of a germline mutation or epimutation in a *MMR* gene, i.e. *MLH1*, *MSH2*, *MSH6* or *PMS2* (Lynch syndrome; LS). The remaining 40% do not show MMR defects and the genetic cause of the familial CRC aggregation is still unknown, having been grouped as familial CRC type X (fCRC-X) [1], [2].

Chromosome telomeres consist of multiple short repeats (TTTAGG) that protect against large-scale genomic rearrangements. Telomeres shorten with cell division, eventually leading to cellular senescence. On rare occasions, cells that aberrantly bypass replicative senescence with critically short telomeres may develop genomic instability and potentially become tumorigenic. In cancer cells, however, as in stem cells, telomerase, the enzyme that adds telomeric repeats to the chromosome ends, is expressed, compensating for telomere erosion and preventing senescence/apoptosis [3]–[5].

Germline mutations in the components of the telomerase complex cause dyskeratosis congenita. Patients with this disorder have short telomeres, which lead to bone marrow failure and increased cancer risk [6]. Likewise, mouse models with telomerase deficiency and short telomeres have high risk of cancer [7]. Recent epidemiological studies have evaluated telomere length measured in peripheral blood DNA as a potential biomarker of cancer risk. Several studies have reported associations between telomere length and cancer risk, although the data are inconsistent among studies and tumor types [8]. In CRC studies, contradictory results have been observed, apparently due to differences in study population, study design, analytical approach, sample size, or exposure to environmental factors [9]–[15].

Regarding hereditary CRC, our group recently reported that cancer-affected *MMR* gene mutation carriers had shorter telomeres and showed faster telomere attrition with age, measured in blood, than controls and cancer-free *MMR* gene mutation carriers [16]. Nevertheless, the role of telomere length as cancer risk modifier in LS could not be asserted since it had been argued that the shortened telomeres observed in retrospectively collected samples from cancer-affected individuals might be a consequence of the disease [8], [14]. However, the fact that cancer-free mutation carriers had longer telomeres than cancer-free controls provided additional evidence in support to the hypothesis that telomere length might act as a cancer risk modifier in LS [16].

Here we report the first study of the behavior of blood telomere length in MMR-proficient hereditary non-polyposis CRC, i.e. fCRC-X, and compare it to the behavior observed in controls and in hereditary non-polyposis CRC with a MMR defect, i.e. LS (previously published [16]).

## Materials and Methods

### Ethics Statement

Written informed consent was obtained from all subjects. The study was approved by the Ethics Committee of IDIBELL (ref. PR221/09).

### Study Participants

A total of 114 individuals, 57 cancer-affected and 57 cancer-free, from 34 fCRC-X families were included in the study. These families fulfilled the Amsterdam criteria but did not show MMR defects (microsatellite instability or loss of expression of the MMR proteins MLH1, MSH2, MSH6 and PMS2). In all, 76.5% (26/34) of the families fulfilled Amsterdam I criteria and 23.5% (8/34) Amsterdam II. For comparative purposes, previously published telomere length data from individuals belonging to LS families and from controls were included in the analyses [16]. Familial CRC-X, LS and control samples were all of Caucasian origin and recruited from the same homogeneous population (the Spanish region of Catalonia) through the Hereditary Cancer Program of the Catalan Institute of Oncology, IDIBELL. Table 1 shows the characteristics of the fCRC-X cases studied, comparing them to the LS individuals and controls previously analyzed [16].

**Table 1:** Characteristics of the hereditary non-polyposis CRC and control groups studied. Data on Lynch syndrome families and controls were published previously [16].

	fCRC-X families (n = 34)		Lynch syndrome families (n = 96) *MMR* *gene mutation carriers*		2Controls	3P-value
	1Cancer	Cancer-free	1Cancer	Cancer-free		
N	57	57	144	100	234	
Median age at blood draw(± SD)	56.0 (±13.9)	50.0 (±20.3)	51.5 (±13.4)	35.0 (±11.5)	42.0 (±14.9)	0.037
Sex: n (%)	M: 27 (47.4)	M: 28 (49.1)	M: 74 (51.4)	M: 40 (40.0)	M: 92 (39.3)	0.968
	F: 30 (52.6)	F: 29 (50.9)	F: 70 (48.6)	F: 60 (60.0)	F: 142 (60.7)	
Median age at cancerdiagnosis (± SD)	49.0 (±13.4)	–	43.0 (±12.9)	–	–	0.278

N, number of subjects; SD, standard deviation; M, male; F, female.

1 Cancer: Individuals affected with a LS-associated cancer: CRC and/or cancer of the endometrium, ovary, stomach, small bowel, hepatobiliary tract, pancreas, upper uro-epithelial tract or brain.

2 Controls include non-carriers from LS families (n = 144) and unrelated cancer-free controls (n = 90).

3 Kruskal-Wallis rank sum test.

### Relative Telomere Length Measurement

Relative telomere length (RTL) was assessed using a monochrome multiplex quantitative PCR method [17], under the same conditions as in Segui et al. [16]. Moreover, MMR-proficient hereditary cases were run together with LS cases and controls. In order to rule out artifacts or technical sources of variation, a standard curve was included in each run (384-well plate), each sample was assayed in triplicate, and whenever possible, equal numbers of samples from different clinical groups were run in the same plate. Storage and DNA extraction from peripheral blood were performed at the same facility and using the same extraction methods, as recommended for retrospective telomere length studies [8].

### Statistical Analyses

RTL was adjusted by age based on the telomere length shortening occurring with age in the general population (controls), as previously reported [16], [18]. Differences in age-adjusted telomere lengths were analyzed using the Wilcoxon rank sum test (Mann-Whitney U). The Kruskal-Wallis rank sum test was used to compare the demographic characteristics among groups. All tests were two-sided and p-values below 0.05 were considered statistically significant. The analyses were performed using R statistical software.

## Results

Age-adjusted RTL was evaluated in 57 cancer-affected fCRC-X patients and 57 unaffected individuals from 34 fCRC-X families. The results were compared to the results we published in a previous study of 144 cancer-affected *MMR* gene mutation carriers, 100 unaffected *MMR* gene mutation carriers and 234 controls [16].

**Figure 1:**
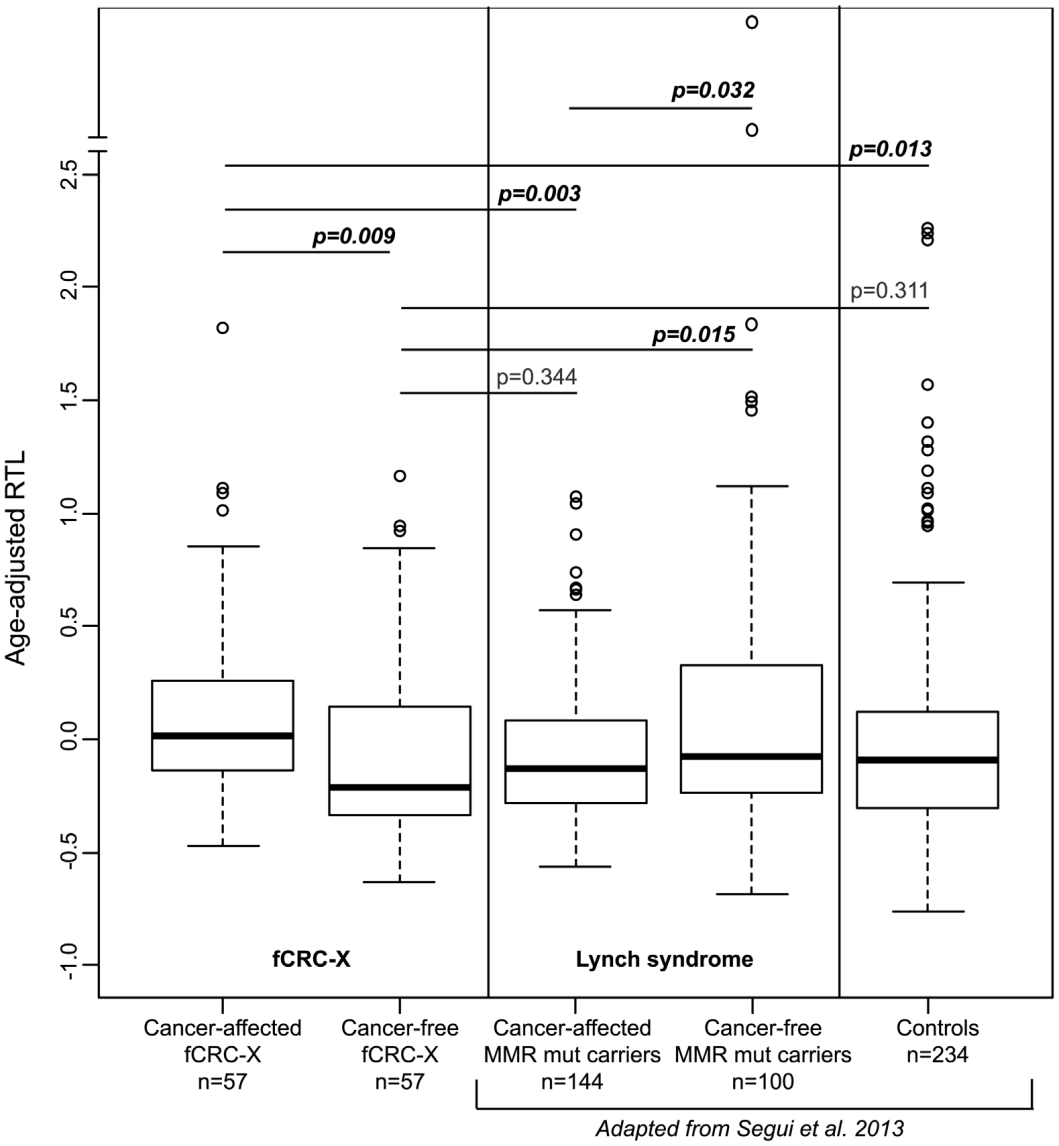
Age-adjusted RTL in subjects belonging to fCRC-X and LS families. The different groups correspond to: cancer-affected fCRC-X cases (median age-adjusted RTL: 0.017); cancer-free individuals from fCRC-X families (median: −0.215); cancer-affected *MMR* gene mutation carriers (median: −0.131); cancer-free *MMR* gene mutation carriers (median: −0.079); and cancer-free controls (median: −0.092). Differences in age-adjusted RTL were analyzed using the Wilcoxon rank sum test (Mann-Whitney U). The boxes represent the interquartile range of distributions (25^th^ and 75^th^ percentiles); the horizontal lines within the boxes, the medians; and the vertical lines, the 5^th^ and 95^th^ percentiles. Data from LS families and controls were published previously [16].

Cancer-affected fCRC-X cases showed significantly longer telomeres than unaffected fCRC-X individuals (p = 0.009) and cancer-free controls (p = 0.013) (Fig. 1). These results were in sharp contrast to those previously observed in LS, where telomere lengths detected in cancer-affected *MMR* gene mutation carriers were shorter than those in unaffected mutation carriers (p = 0.032) [16]. On the other hand, cancer-free fCRC-X cases showed shorter telomeres than cancer-free *MMR* gene mutation carriers (p = 0.015), but of similar length to controls (Fig. 1).

## Discussion

We found that longer telomeres are associated with cancer in fCRC-X. Moreover, based on data previously published by our group [16], MMR-deficient and -proficient non-polyposis hereditary cases show distinct patterns of blood telomere length, suggesting that the status of the MMR system is key in defining telomere length in hereditary cases.

Several epidemiological studies have investigated the association of telomere length with CRC risk in the general population, producing conflicting results [19]. Evidence from retrospective studies indicates that telomeres in peripheral blood cells are shorter in CRC cases than controls. However, this association has not been replicated in prospective studies, suggesting that the association between short telomeres and CRC in retrospective studies is somehow the result of disease, treatment or differential survival rather than the cause (effect of reverse causation) [14]. Regarding prospective studies, no association was found in three relatively small studies (134–191 cases vs. 306–406 controls) [9]–[11]. However, more recently, larger prospective studies found that longer telomeres were associated with higher CRC risk (cases/controls = 2,157/3,921) [14], or that both long and short telomeres increased CRC risk (cases/controls = 441/549) [15].

Despite the retrospective nature of our study, we found that cancer-affected fCRC-X individuals had longer telomeres than cancer-free members of the same type X families, controls, and cancer-affected members of LS families (*MMR* gene mutation carriers). Therefore, longer telomeres are associated with cancer risk in MMR-proficient hereditary non-polyposis CRC, as observed in large prospective population-based CRC series [14], [15]. Further studies assessing telomere length before and after cancer diagnosis in fCRC-X cases will be crucial to demonstrate the precise effect of cancer on blood telomere length in this group of patients.

Previous evidence supports the hypothesis that long telomeres might increase cancer risk: first, long telomeres may delay cellular senescence and apoptosis, increasing the chance that genetic abnormalities will accumulate [5], [20]; secondly, a subset of colorectal tumors has longer telomeres than the adjacent non-tumor colon mucosa [21]. Telomere length assessment in fCRC-X tumors will provide additional insight into whether MMR-proficient hereditary tumors also have elongated telomeres. Similarly, knowing the levels of telomerase expression or of enzymatic activity in peripheral blood and tumor samples might aid the design of specific therapeutic and/or preventive approaches for fCRC-X patients in the future [22].

Our findings, together with those observed in large population-based CRC series [14], [15], suggest that longer telomeres are a risk factor for hereditary and sporadic MMR-proficient CRC. Following this observation, it is plausible to hypothesize that longer telomeres also act as modifiers of the age of onset of cancer and/or polyposis in other MMR-proficient CRC syndromes.

In conclusion, in contrast to in the observations for LS but in line with the results of large CRC prospective studies, elongated telomeres are associated with increased cancer risk in Amsterdam-positive MMR-proficient hereditary non-polyposis CRC.
